# Exploring Product Innovation and Consumer Attitudes in Molecular Gastronomy: Marketing Insights for the Gourmet Food Industry

**DOI:** 10.3390/foods14020209

**Published:** 2025-01-10

**Authors:** Min-Yen Chang, Chiao-Xuan Huang, I-Kai Lin, Han-Shen Chen

**Affiliations:** 1 Department of Accounting, Jiaxing University, Jiaxing 314001, China; mingyen0223@zjxu.edu.cn; 2 Department of Health Industry Technology Management, Chung Shan Medical University, Taichung 40201, Taiwan; a0909182012@gmail.com (C.-X.H.); linken910428@gmail.com (I.-K.L.); 3 Department of Medical Management, Chung Shan Medical University Hospital, Taichung 40201, Taiwan

**Keywords:** molecular gastronomy, utilitarian value, hedonic value, product novelty, sensory stimulation

## Abstract

The increasing popularity of social media and the growth of gourmet food culture have elevated molecular gastronomy as a unique dining experience that enhances consumers’ perceptions of value through innovative food presentation and sensory marketing strategies. This study investigates consumer acceptance of molecular gastronomy by utilizing the value–attitude–behavior (VAB) model and the Theory of Planned Behavior (TPB). We examine the interplay between utilitarian and hedonic values, product innovation, and sensory stimulation to understand consumer reactions and attitudes toward molecular gastronomy. Through convenience sampling, we surveyed individuals who had experienced molecular gastronomy, collecting 407 valid responses, with a response rate of 95.3%. Our findings reveal that both utilitarian and hedonic values significantly influence consumers’ attitudes (β = 0.635, *p* < 0.01; β = 0.750, *p* < 0.01). Attitudes, perceived behavioral control, and sensory stimulation play crucial roles in shaping behavioral intentions (β = 0.770, *p* < 0.01; β = 0.719, *p* < 0.01; β = 0.791, *p* < 0.01). Although subjective norms and product novelty also have positive effects on intentions, their impact is less significant (β = 0.511, *p* < 0.01; β = 0.416, *p* < 0.01). These insights suggest that practitioners in the dining industry should prioritize utilitarian value, hedonic appeal, consumer attitudes, perceived behavioral control, and sensory experiences when creating and marketing molecular gastronomy dishes. This research not only enhances our understanding of consumer behavior within this innovative culinary domain but also offers practical strategies for boosting market acceptance and engagement with such avant-garde dining experiences.

## 1. Introduction

In recent years, the rapid growth of social media has resulted in an increased number of individuals sharing their culinary experiences online, thereby promoting a refined dining culture [1]. Notable examples of this trend include the restaurant “Kitchen Theory”, which introduced the Náttúra concept, “Le Petit Chef”, known for its use of 3D projection technology, and “Elan Vital”, which combines 3D light sculptures and installation art to elevate the dining experience. These establishments exemplify the potential of molecular gastronomy to enhance sensory stimulation through innovative cooking techniques, thus creating unique dining experiences that motivate further research in this area.

Originally proposed by French chemist Hervé This and Hungarian physicist Kürti Miklós in 1988, molecular gastronomy has undergone significant technological advancements in terms of production methods, presentation, and flavor profiles [2]. This culinary style challenges the traditional norms of cooking by focusing on the intrinsic properties of ingredients and accounting for variables such as cooking time, temperature, and the interaction of different components. Research indicates that highly novel products possess distinct functional characteristics that can indirectly enhance consumers’ behavioral intentions [3,4]. Recognizing the distinctions between molecular gastronomy and traditional cuisine, this study incorporates product novelty as a key variable in providing a comprehensive analysis of consumer perceptions.

Molecular gastronomy attracts consumers through distinctive cooking methods and experiences [5]. Studies have shown that consumers with utilitarian or hedonic value are likely to exhibit increased behavioral intentions based on enhanced food quality or enjoyable experiences offered by restaurants [6]. To explore the relationship between consumer values and attitudes toward molecular gastronomy, this study applies the value–attitude–behavior model (VAB). The VAB model is widely used to analyze consumer attitudes toward innovative food products. For instance, Lee et al. [7] demonstrated that both utilitarian and hedonic values significantly influence consumer attitudes toward 3D-printed foods, with hedonic value exerting a more substantial effect. Similarly, Saini et al. [8] investigated how green values affect attitudes toward plant-based meat, whereas Brouwer et al. [9] explored the impact of anti-species discrimination values on attitudes toward vegan diets. However, existing research has not adequately addressed the unique elements of molecular gastronomy, such as price, time commitment, and entertainment value, which may shape consumer attitudes. This study aimed to fill this gap by utilizing the VAB model.

Additionally, the TPB has been widely applied to examine consumer behavioral intentions across various contexts, including organic foods [10], ugly foods [11], insect-based foods [12], and cultured meat [13]. For example, Tai et al. [14] used the TPB framework to analyze consumers’ intentions regarding edible offal from cattle, whereas Tomić Maksan and Jelić [15] investigated Croatian consumers’ behavioral intentions toward domestically produced chips. Mopendo et al. [16] employed TPB to explore the behavioral intentions of individuals in southwestern Congo regarding edible insects. The findings of these studies consistently indicate that factors such as attitudes, subjective norms, and perceived behavioral control significantly influence consumers’ behavioral intentions.

Therefore, although molecular gastronomy has gradually become a research hotspot owing to its innovative culinary techniques and sensory experiences, existing studies have primarily focused on its technical aspects. Research on how sensory stimulation and product novelty influence consumer behavioral intentions remains limited, resulting in a lack of systematic theoretical exploration in this field. This study aimed to utilize the VAB and TPB models as core frameworks, incorporating sensory stimulation and product novelty, to address the research gap in molecular gastronomy and explore their effects on consumer behavioral intentions. The combination of these two models allows for a comprehensive explanation of consumer behavioral intentions from different perspectives. The VAB model provides a profound understanding of value-driven factors, while the TPB complements this by focusing on the psychological processes and social factors that influence behavioral intentions. By integrating both models, this study can more comprehensively explore the impact of sensory stimulation, product novelty, and other influencing factors on consumer attitudes and behavior and examine how value-driven factors and psychological processes interact, offering a diverse and comprehensive perspective on consumer behavior in the field of molecular gastronomy. The results are expected to offer valuable insights into food and beverage businesses in terms of meal development and market positioning.

The second part of this study provides a detailed literature review and develops hypotheses regarding the variables in the VAB and TPB models. The third part describes the research framework, design, sample collection, and statistical analyses. The fourth part presents the overall data analysis. The fifth part provides a detailed discussion of the results. The sixth section summarizes the research findings and presents theoretical and practical implications.

## 2. Literature Review and Hypothesis Development

### 2.1. Value–Attitude–Behavior Model

The value–attitude–behavior model (VAB) introduced by Homer and Kahle [17] serves as a foundational framework elucidating how values influence consumers’ attitudes, which in turn shape their behavioral intentions. This study focuses on two primary types of values, utilitarian value and hedonic value, derived from the principles of molecular gastronomy and relevant literature.

#### 2.1.1. Utilitarian Value

Consumers who prioritize utilitarian values tend to be driven by cognitive and goal-directed behaviors. They generally focus on product attributes such as convenience, cost, and functionality, perceiving purchasing as a task-oriented process [18]. Studies by Sadiq et al. [19] and Shin et al. [20] highlight that organic foods characterized by utilitarian values, such as healthiness and the absence of chemicals, can positively influence consumer attitudes. Additionally, food service options such as food trucks emphasize convenience, further reinforcing this relationship.

Molecular gastronomy, which is often marked by complex preparation processes and visually distinct presentations [21], offers several utilitarian characteristics. Factors such as convenience of consumption, dining duration, portion size, health benefits, and consumers’ willingness to pay are crucial in shaping consumers’ perceptions. Thus, this research posits that these utilitarian aspects will significantly enhance consumers’ attitudes toward molecular gastronomy, leading to the following hypothesis:

**Hypothesis** **1 (H1).** *Utilitarian value (e.g., convenience, price, and functionality) has a significant positive impact on consumers’ attitudes toward molecular gastronomy.*

#### 2.1.2. Hedonic Value

In contrast, consumers motivated by hedonic value are primarily influenced by the feelings and enjoyment derived from consumption, with purchases based on the entertainment, relaxation, or personal significance provided by products or services [18]. Hepola et al. [22] articulate that when consumers engage in hedonic consumption, their psychological involvement deepens, aligning with Valesi et al. [23], who find that pleasure-seeking consumers exhibit greater acceptance of novel food items.

Molecular gastronomy aims to innovate by concocting unexpected products and food combinations and employing creative cooking techniques and advanced technology to surprise consumers [5]. Given these attributes, we hypothesize that hedonic value significantly enhances consumers’ attitudes toward molecular gastronomy, culminating in the following hypothesis:

**Hypothesis** **2 (H2).** *Hedonic value (e.g., pleasure, satisfaction, and entertainment) has a significant positive impact on consumers’ attitudes toward molecular gastronomy.*

### 2.2. Theory of Planned Behavior

The Theory of Planned Behavior (TPB), articulated by Ajzen [24], is a well-established social psychological theory that provides insights into human actions through antecedents, such as attitudes, subjective norms, and perceived behavioral control, all of which culminate in intentions and subsequent behaviors. This study expands upon TPB by integrating additional variables, namely, utilitarian value, hedonic value, product novelty, and sensory stimulation.

#### 2.2.1. Attitude

Attitudes reflect enduring evaluations of people, objects, or events and serve as predictors of behavioral tendencies [24]. Research indicates that favorable attitudes toward innovative functional foods, including beef offal and red palm oil additives, can significantly influence behavioral intentions [14,25]. Thus, a positive attitude toward molecular gastronomy, which encompasses safety, taste, quality, and waste reduction, is expected to enhance consumers’ behavioral intentions.

#### 2.2.2. Subjective Norm

According to Singh et al. [26], subjective norms represent perceived social pressure to engage in specific behaviors. These norms can be shaped by both formal (e.g., regulations) and informal sources (e.g., family and friends) [15]. A stronger subjective norm typically correlates with a heightened intention to perform a behavior [27]. However, Joo et al. [28] observed that subjective norms do not significantly influence the behavioral intentions of Korean consumers regarding indoor farming restaurants. Given the novelty of molecular gastronomy, subjective norms may crucially inform behavioral intentions, as consumers often rely on the evaluations of others in unfamiliar contexts.

#### 2.2.3. Perceived Behavioral Control

Ajzen [24] described perceived behavioral control as the degree to which individuals feel that they can execute a behavior influenced by past experiences and anticipated barriers. Research indicates that perceived behavioral control significantly affects intentions regarding novel food categories such as insects and organic produce [29]. Regarding willingness to purchase cultivated meat, perceived behavioral control significantly influences factors such as accessibility and pricing [30]. In the context of molecular gastronomy, this control may encompass factors such as willingness to pay, perceived accessibility, and duration of enjoyment. Higher perceived behavioral control may lead to increased engagement with molecular gastronomy.

#### 2.2.4. Behavioral Intention

Behavioral intention represents the degree of intent to partake in a specific behavior, reflecting psychological commitment to action during decision-making [31]. As measuring actual behaviors can be problematic, behavioral intention often serves as an effective surrogate [32]. By synthesizing insights from the literature, it is evident that consumers’ behavioral intentions toward molecular gastronomy are influenced by their attitudes, subjective norms, and perceived behavioral control. Consequently, this study proposes the following hypotheses:

**Hypothesis** **3 (H3).** *Consumers’ attitudes significantly positively influence their behavioral intentions toward molecular gastronomy.*

**Hypothesis** **4 (H4).** *Consumers’ subjective norms significantly and positively influence their behavioral intentions toward molecular gastronomy.*

**Hypothesis** **5 (H5).** *Consumers’ perceived behavioral control significantly and positively influences their behavioral intentions toward molecular gastronomy.*

### 2.3. Product Novelty

Innovation is often regarded as a catalyst for creating new products and ideas [33]. The novelty of a product can bridge the gap between functionality and esthetic appeal [3]. Product novelty plays a pivotal role in shaping consumers’ behavioral intentions. For instance, Wang et al. [4] found that service robot novelty affects exploratory consumption behavior. Although many consumers demonstrate apprehension toward novel foods [34], this study contends that molecular gastronomy, exemplified by techniques such as foaming, can capture consumers seeking unique culinary experiences. Therefore, this study investigates how distinctive styles of dishes and dining experiences associated with molecular gastronomy impact consumers’ behavioral intentions, leading to the following hypothesis:

**Hypothesis** **6 (H6).** *The novelty of molecular gastronomy significantly and positively influences consumers’ behavioral intentions.*

### 2.4. Sensory Stimulation

As noted by Schmitt [35], sensory stimulation affects consumers’ expectations and reactions through their visual, auditory, tactile, gustatory, and olfactory senses [36], which in turn affect their preferences and intentions to consume food [37]. Wendin and Nyberg [38] highlighted that the sensory quality of food is crucial for consumer acceptance, particularly in the context of innovative food options, such as insect-based dishes. This study sought to evaluate how sensory stimulation factors, including visual appeal, texture, aroma, taste, and even sounds associated with molecular gastronomy, influence consumers’ behavioral intentions. Consequently, we propose the following hypothesis:

**Hypothesis** **7 (H7).** *Sensory stimulation of molecular gastronomy significantly and positively influences consumers’ behavioral intentions.*

## 3. Research Methodology

### 3.1. Research Framework

**Figure 1:**
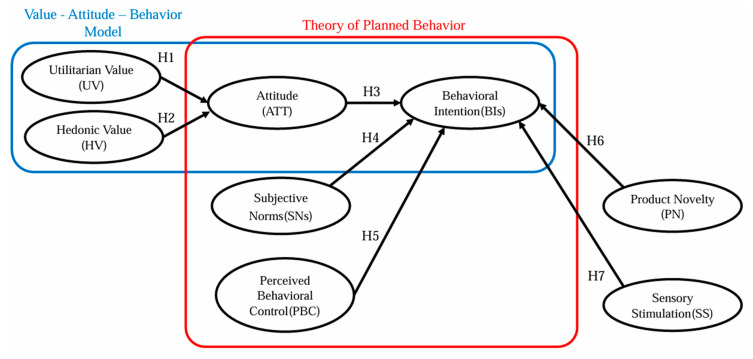
Conceptual framework and hypotheses of the study. Note: blue, value–attitude–behavior model; red, theory of planned behavior.

This study aims to integrate the VAB model with the TPB to establish a robust research framework to enhance our understanding of consumer behavior. By incorporating two key dimensions, product novelty and sensory stimuli, this framework offers a multifaceted perspective on consumer motivations. Building on Wang et al.’s [4] exploration of product novelty and Guedes et al.’s [37] analysis of sensory stimuli, this approach seeks to expand consumer behavior considerations, providing a more comprehensive and nuanced understanding of consumer behavior patterns (detailed in Figure 1).

### 3.2. Research Questionnaire Design

The questionnaire designed for this study comprised three main sections, each aimed at analyzing the determining factors influencing Taiwanese consumers’ behavioral intentions toward molecular medicine.

#### 3.2.1. Conceptual Introduction

Recognizing that molecular cuisine is an innovative food category unfamiliar to many respondents, the first section of the questionnaire served as a conceptual introduction. This section is intended to provide respondents with sufficient background information, thereby mitigating potential ambiguities surrounding the concept of molecular cuisine. By enhancing the respondents’ understanding, this measure aims to reduce the likelihood of random or uninformed choices during the completion of the subsequent sections of the questionnaire.

#### 3.2.2. Psychological Constructs Measurement

The second section of the questionnaire was predicated on the VAB model and the TPB. It includes questions targeting critical psychological constructs, such as utilitarian value, hedonic value, attitude, subjective norm, perceived behavioral control, and behavioral intention. The items corresponding to utilitarian and hedonic values were meticulously adjusted and refined based on prior research conducted by Sadiq et al. [19], Shin et al. [20], Valesi et al. [23], and Hepola et al. [22]. Furthermore, the variables and their associated question items relevant to the TPB are synthesized from findings reported in studies by Sabbagh et al. [25], Tai et al. [14], Tomić Maksan and Jelić [15], Joo et al. [28], Mopendo et al. [16], Siripipatthanakul et al. [30], Lin and Roberts [39], and Hamid and Bano [32].

#### 3.2.3. Adding Additional Variables

To attain a more holistic view of the factors influencing consumer behavioral intentions, this study incorporated two additional variables: product novelty and sensory stimuli. The question design and adjustments for these variables are grounded in the relevant research by Ruiz-Pastor et al. [33], Wang et al. [4], Ruiz-Capillas et al. [36], and Wendin and Nyberg [38].

#### 3.2.4. Scale Design and Demographic Data Collection

Each of the aforementioned variables was assessed using a seven-point Likert scale, enabling respondents to express their agreement ranging from “strongly disagree” (1) to “strongly agree” (7). This scale design facilitates the accurate capture of respondents’ perceptions and evaluations of specific attributes. Additionally, the third section gathered respondents’ demographic information, including gender, age, education level, and personal monthly income. These demographic data are essential for contextualizing the analysis and ensuring the robustness of the interpretive framework. Through this structured questionnaire design, this study aims to identify the key factors influencing Taiwanese consumers’ behavioral intentions toward molecular medicine.

### 3.3. Sample Collection and Methods of Data Analysis

This study employs a quantitative research method that utilizes a deductive approach to test hypotheses derived from existing theories and employs a convenience sampling method that allows for effective and easy access to a large number of participants within a limited timeframe. The questionnaire was distributed through online platforms (e.g., Facebook and Instagram), and participants accessed the survey by clicking on a shared link rather than receiving it directly via email sent to specific groups. The inclusion criteria for the sample were that participants had to be at least 18 years old and of Taiwanese nationality. This approach not only facilitated the rapid collection of data but also enabled the sample to reflect a diverse range of backgrounds. To ensure the anonymity of the respondents, the survey was designed for anonymous completion, with the collected data restricted solely to academic research purposes and not intended for public disclosure. Specifically targeting consumers familiar with molecular cuisine, the questionnaire aimed to gather pertinent data that facilitated subsequent analyses aligned with the research objectives and hypothesis verification.

Data analysis was performed using IBM SPSS Statistics 18 and AMOS 27 for statistical evaluation. The analysis methods included descriptive statistics, such as frequency distribution tables, percentages, means, and standard deviations. Reliability and validity analyses were conducted to ensure the integrity of the measurement instruments. The primary analytic technique, Structural Equation Modeling (SEM), is utilized to quantify the relationships between constructs such as utilitarian value, hedonic value, attitude, subjective norms, perceived behavioral control, product novelty, sensory stimuli, and consumer characteristics. This analysis will further elucidate causal relationships and the overall model fit concerning the proposed hypothesis model, thereby verifying the research hypotheses posited in this study.

## 4. Analysis and Results

### 4.1. Demographic Analysis

This study utilized a convenience sampling method, distributing a questionnaire through an online platform, which resulted in the collection of 427 responses. After the screening process, 407 responses were validated, resulting in a valid response rate of 95.3%. All participants were Taiwanese. In terms of gender, 39.6% of respondents were male, while 60.4% were female, indicating a higher participation rate among females.

The age distribution revealed that the predominant demographic group was the 31–40 age range, which accounted for 45.5% of the respondents. This was followed by the 18–30 and 41–50 age groups, accounting for 23.6% each. Collectively, the youth and middle-aged groups (ages 21–50) comprised 91.2% of the sample.

Regarding educational background, 83.3% of the respondents held a college degree or higher, reflecting a generally elevated educational level within the sample population.

In terms of personal average monthly income, 39.3% of the respondents reported earnings between TWD 20,001 and TWD 40,000, representing the largest income group. Additionally, 34.2% of the respondents fell into the income bracket of TWD 40,001 to TWD 60,000. Notably, respondents earning above the national average income of TWD 48,422 were a minority. Table 1 presents the relevant demographic variables.

### 4.2. Measurement Model: Reliability and Validity

To ensure the robustness of the reliability and validity of this study’s findings, statistical software SPSS 18.0 and structural equation modeling software AMOS 27.0 were employed for comprehensive data analysis. The analytical framework encompasses eight core dimensions: utilitarian value, hedonic value, attitude, subjective norms, perceived behavioral control, behavioral intention, product novelty, and sensory stimulation. Preliminary analysis in SPSS focused on evaluating standardized factor loadings across these dimensions, along with composite reliability (CR), average variance extracted (AVE), and Cronbach’s α coefficients, with the results detailed in Table 2.

Items with factor loadings below 0.5 were excluded from further analysis. Consequently, the following items were removed: question 4 on subjective norm, questions 4 and 5 on perceived behavioral control, and questions 3 and 5 on utilitarian value. The composite reliability (CR) values for the dimensions ranged from 0.857 to 0.946, indicating a high level of internal consistency among the dimensions. Additionally, to ensure that the measurement variables were effectively represented by the constructs, the average variance extracted (AVE) values ranged from 0.667 to 0.814, demonstrating good explanatory power. A Cronbach’s α coefficient greater than 0.7 is considered indicative of high reliability, and in this study, all dimensions exceeded this threshold, confirming the reliability of the questionnaire data. Furthermore, all factor loading values exceeded the minimum threshold of 0.5, confirming the validity of the data used in this research.

Discriminant validity in structural equation modeling refers to the assessment of two distinct concepts and an analysis of their correlation outcomes. A minimal correlation suggests that there is discriminant validity between these concepts. Hair et al. [40] state that the correlation coefficient of two different concepts must be lower than the square root of the average variance extracted (AVE) for each concept. In Table 3, a comparison is made between the correlation coefficients and the square roots of the AVE for all dimensions examined in this study. The square root values of the AVE for each dimension exceeded the correlation coefficients between pairs of dimensions, which aligns with the standards outlined by Hair et al. [40] and confirms the presence of discriminant validity among the dimensions in this study. From the assessment of the measurement model, it can be inferred that the model utilized in this research demonstrated solid internal and external validity.

### 4.3. Model Fit Test

For model fit testing, we conducted a confirmatory factor analysis (CFA) using the maximum likelihood estimation method (MLE). According to the standards proposed by Bagozzi and Yi [41], the evaluation results indicated that although a few fit indices did not fully meet the recommended values, the overall fit indices demonstrated a high degree of consistency between the research data and proposed theoretical model. The specific values of the fit indices are presented in Table 4.

### 4.4. Overall Model Path Analysis

To examine the relationships within the VBA model, linear regression analysis was performed using SPSS software (version 18.0) to examine relationships within the VBA model.

#### 4.4.1. Impact of Utilitarian and Hedonic Values

The first analysis focuses on the effect of utilitarian value on consumers’ attitudes toward molecular gastronomy. The results confirmed Hypothesis “H1: Utilitarian value has a significant positive impact on consumers’ attitudes toward molecular gastronomy” (β = 0.635, *p* < 0.01), supporting the validity of this hypothesis. Similarly, hedonic value also demonstrated a significant positive impact on attitudes, corroborating hypothesis “H2: Hedonic value has a significant positive impact on consumers’ attitudes toward molecular gastronomy” (β = 0.750, *p* < 0.01).

#### 4.4.2. Application of the Theory of Planned Behavior

Further exploration based on the TPB encompassed three variables. First, attitudes were found to significantly influence behavioral intentions regarding molecular gastronomy, supporting hypothesis “H3: Consumers’ attitudes have a significant positive impact on their behavioral intentions toward molecular gastronomy” (β = 0.770, *p* < 0.01). Second, subjective norms also demonstrated a statistically significant positive impact on behavioral intentions, in alignment with hypothesis “H4: Consumers’ subjective norms have a significant positive impact on their behavioral intentions toward molecular gastronomy” (β = 0.511, *p* < 0.01). Finally, perceived behavioral control significantly affected behavioral intentions, confirming the hypothesis “H5: Consumers’ perceived behavioral control has a significant positive impact on their behavioral intentions toward molecular gastronomy” (β = 0.719, *p* < 0.01).

#### 4.4.3. Examination of Product Novelty and Sensory Stimulation

In examining the additional variables of product novelty and sensory stimulation, results revealed that the novelty of molecular gastronomy significantly influences behavioral intentions, supporting the hypothesis “H6: The novelty of molecular gastronomy significantly positively influences consumers’ behavioral intentions” (β = 0.416, *p* < 0.01). Concurrently, sensory stimulation from molecular gastronomy also exhibited a significant positive effect on behavioral intentions, affirming the hypothesis “H7: The sensory stimulation from molecular gastronomy significantly positively influences consumers’ behavioral intentions” (β = 0.791, *p* < 0.01).

#### 4.4.4. Path Analysis Interpretation

**Figure 2:**
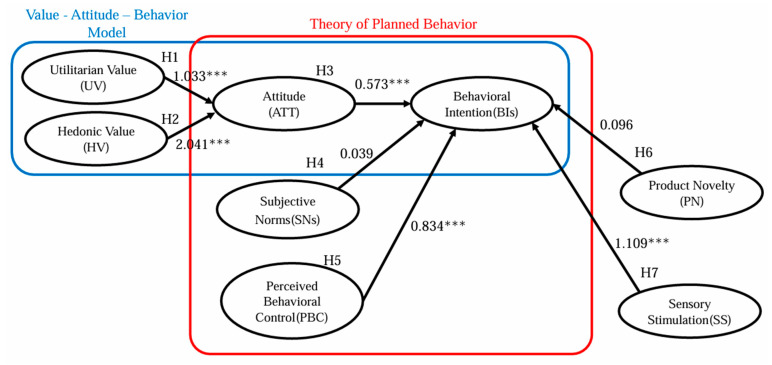
Structural equation modeling diagram. Note: blue, value–attitude–behavior model; red, theory of planned behavior. *** *p* < 0.001.

The empirical findings offer critical insights into the factors that drive consumer acceptance and intention regarding molecular gastronomy. Structural Equation Modeling (SEM) was employed for path analysis to validate the hypothesized relationships, and the resulting path coefficients illustrated the strength and directionality of these relationships, as depicted in Figure 2.

In summary, both utilitarian and hedonic values have a significantly positive influence on consumer attitudes. Among the three TPB variables, only attitude and perceived behavioral control demonstrated significant positive impacts on behavioral intentions, underscoring their critical roles in consumers’ decision-making processes. Notably, while the influence of product novelty on behavioral intention did not achieve statistical significance, indicating that it may not be a crucial factor influencing behavioral intentions in the context of this study, sensory stimulation emerged as a substantial facilitator in shaping these intentions. The comprehensive results of path analysis are presented in Table 5.

## 5. Discussion

This study is centered on the VAB and TPB, aiming to analyze the impact of product novelty and sensory stimulation on consumer behavioral intentions toward molecular cuisine. The results provide essential insights that can guide meal development and restaurant market positioning.

### 5.1. Impact of Utilitarian and Hedonic Values

The findings reveal that both utilitarian and hedonic values exert a positive and significant influence on consumer attitudes. This aligns with the work of Sadiq et al. [19] and Hepola et al. [22], which highlights that molecular cuisine embodies dual utilitarian and hedonic characteristics. Notably, the impact of hedonic value (β = 0.750) on consumer attitudes surpasses that of utilitarian value (β = 0.635). This trend correlates with the growing gourmet dining culture, wherein consumers increasingly prioritize the overall dining experience over functionality. Additionally, Kelly [42] and Yuan et al. [43] emphasize that pleasurable and engaging dining experiences enhance hedonic value, subsequently boosting consumers’ intentions to continue utilizing such services. Thus, the results of this study further substantiate the notion that hedonic considerations significantly shape consumer behavior in the context of molecular medicine. This also suggests that in addition to enhancing meal delivery speed and convenience, businesses should focus on improving the pleasurable and entertaining aspects of their dishes and services to foster more positive consumer attitudes.

### 5.2. Theory of Planned Behavior (TPB)

Although attitude, subjective norm, and perceived behavioral control demonstrated positive significance in the regression analysis, the path analysis revealed that only attitude and perceived behavioral control had a significant positive impact on behavioral intentions. The subjective norm, although positively correlated, was not statistically significant. This finding diverges from the conclusions of Maksan and Jelić [15] regarding purchase intentions for potato chips, a product characterized by affordability and widespread acceptance. Consequently, consumers have fewer reference points and evaluations when considering molecular medicines. Furthermore, the growing influence of social media may exacerbate this situation; negative experiences shared online can generate social pressure [44], potentially diminishing behavioral intentions toward molecular cuisine.

Additionally, the high price of molecular cuisine complicates the role of subjective norms. When the product price is higher than that of traditional products, consumers tend to make more cautious decisions and seek validation from trusted sources [45,46]. In the case of molecular cuisine, the lack of strong subjective norms, due to fluctuating cultural acceptance, may hinder consumers from overcoming the perceived risk associated with high prices. Furthermore, consumers’ low familiarity with molecular cuisine exacerbates this issue as they struggle to assess the value of such products based on past experiences or social circles. Thus, it is plausible that the high prices, low consumer familiarity, and pervasive influence of social media play critical roles in lessening the impact of subjective norms on behavioral intentions in this specific culinary context.

### 5.3. The Impact of Product Novelty on Behavioral Intentions

According to our path analysis, although molecular cuisine has garnered attention for its innovative cooking techniques and unique presentations, its impact on consumer behavioral intentions is positive but not statistically significant. Previous studies have shown that novelty and desire for new experiences play an important role in driving consumer behavior [47]. However, the results of this study are inconsistent with these findings, suggesting that the impact of these driving factors may be more complex in the context of molecular cuisine and may be influenced by various factors. This inconsistency may stem from potential issues in the design of the questionnaire, particularly the ambiguity of the term ‘novelty’ [48]. During the questionnaire design, respondents may have different interpretations of “novelty”, with some consumers possibly viewing molecular cuisine as merely over-decorated traditional dishes or an extension of past dining experiences rather than seeing it as a truly innovative experience. This may have led to a lack of significant results regarding the impact of novelty on consumer behavioral intentions.

Furthermore, food neophobia is another important factor affecting consumer acceptance of new types of cuisine. Research has shown that food neophobia plays a significant role in consumers’ acceptance of unfamiliar foods, which, in turn, limits their openness to molecular cuisine [24,49,50]. This psychological barrier often results in more conservative attitudes toward molecular cuisine, thereby weakening the impact of innovation on consumers’ behavioral intentions. Therefore, despite the significant novelty of molecular cuisine, if consumers hold reservations about these new experiences, novelty may not translate into actual behavioral intentions.

### 5.4. The Impact of Sensory Stimulation on Behavioral Intentions

Our findings indicate that the sensory stimulation offered by molecular cuisine significantly positively influences consumer behavioral intentions, highlighting the distinctive ability of this cuisine to engage the senses and elevate consumer preferences. This aligns with the research of Guedes et al. [37], Ruiz-Capillas et al. [36], and Wendin and Nyberg [38], which reaffirms the importance of sensory experiences in culinary contexts. Notably, among the sensory attributes assessed, taste received the highest average score (5.07), closely followed by visual appeal (4.97). The prominence of appearance as an indicator of food quality underscores its potential as a critical factor for consumer decision-making [51]. Notably, the dessert shop MINIMAL employs liquid nitrogen techniques to create visually captivating ice cream presentations paired with rich flavors, exemplifying the potential for sensory engagement. This indicates that businesses should prioritize innovative approaches to enhance both taste and visual appeal in their offerings to attract prospective consumers. Therefore, stakeholders in the food industry should focus on enhancing the uniqueness of flavors and visual presentations during meal development. Techniques such as emulsification to create visually appealing foamy textures combined with layered flavors could strategically attract and retain potential consumers in the market for molecular cuisines.

By thoughtfully addressing these areas, food industry professionals can leverage the insights gained from this study to create engaging dining experiences that entice consumers, thereby fostering broader acceptance and ongoing interest in molecular cuisine.

## 6. Conclusions

### 6.1. Research Conclusions

Based on the above results and discussion, we reached the following conclusions. First, within the VAB framework, both utilitarian and hedonic values exert positive and significant influences on consumer attitudes, with hedonic values showing a greater degree of impact. 

Second, the application of TPB indicates that only attitude and perceived behavioral control significantly impact consumers’ behavioral intentions. The lack of significant influence from subjective norms may be attributed to the high cost and limited popularity of molecular cuisine, which hinders consumers’ ability to draw adequate reference points for evaluation. 

Third, although product novelty was found to have a positive impact on behavioral intentions, this effect was not significant. This study hypothesized that ambiguous questionnaire items may have led to varied interpretations of novelty among respondents. Additionally, food neophobia—reluctance to try unfamiliar foods—could be an important factor affecting consumers’ willingness to appreciate molecular cuisine.

Finally, the study underscores that sensory stimulation plays a significant role in influencing consumers’ behavioral intentions, with taste and visual elements identified as the most critical factors. 

### 6.2. Management Recommendations

From a managerial perspective, the findings indicate that future entrepreneurs and existing businesses in the food industry should strategically balance the dual pursuit of utilitarian and hedonic value in product development and marketing strategies. This can include creating innovative dishes that not only meet functional needs such as convenience and health but also provide rich sensory experiences to enhance consumer enjoyment. For instance, incorporating visually appealing presentations and unique flavor combinations can attract consumers seeking novelty and entertainment.

Additionally, businesses should focus on enhancing consumers’ positive attitudes and perceived behavioral control by providing clear information about the preparation process and the sustainability of molecular cuisine. Educational initiatives and experiential dining activities can help consumers overcome their unfamiliarity with molecular cuisine, thereby attracting a broader range of potential customers.

### 6.3. Research Limitations and Future Research Directions

First, the focus of this study was limited to the Taiwanese market, which may restrict the generalizability of the findings to other cultural or geographical contexts. Future studies could benefit from a broader demographic approach that includes consumers from various regions (e.g., other Asian countries) to enhance the applicability of the research outcomes.

Second, this study did not differentiate between specific dietary habits among consumers, such as those preferring whole foods and those accustomed to refined dining experiences. Understanding the nuances of these consumer segments could lead to insights that reflect differing behavioral outcomes. Future research should consider integrating dietary preference as a variable for exploration.

Moreover, while molecular cuisine attracts consumers to seek hedonic value and novelty, it is important to acknowledge the complexity of its appeal. The processes involved in molecular gastronomy often use ingredients such as emulsifiers to achieve desirable presentations and flavors, which may deter health-conscious consumers or those with food neophobia. These attributes can significantly diminish behavioral intentions. Future investigations should include the dimensions of health values and food neophobia to assess how such characteristics influence consumer responses to molecular cuisine.

Additionally, this study employed a convenience sampling method. However, according to Doebel and Frank [52], convenience sampling may fail to ensure sample diversity while promoting theoretical development or reducing research bias, thereby limiting the broad applicability of our findings. Future research could consider using a variety of convenience groups to obtain ’inconvenient’ samples or employing different sampling methods (e.g., stratified sampling) to ensure the representativeness of each subgroup selected based on specific attributes, thus enhancing the generalizability of the overall results.

## Figures and Tables

**Table 1:** Demographic analysis examining the characteristics of a population.

*N* = 407	Item	Population	Percentage (%)
Gender	Male	161	39.6
	Female	246	60.4
Age	18–30 years	96	23.6
	31–40 years	185	45.5
	41–50 years	96	23.6
	51–60 years	25	6.1
	61 years and above	5	1.2
Level of Education	Junior high school or below	9	2.2
	High school/vocational	59	14.5
	College/university	299	73.5
	Master’s or above	40	9.8
Monthly personal income	Less than NTD 20,000 (USD 660) (inclusive)	35	8.6
	NTD 20,001–40,000 (USD 660–1320)	160	39.3
	NTD 40,001–60,000 (USD 1320–1980)	139	34.2
	NTD 60,001–80,000 (USD 1980–2640)	47	11.5
	NTD 80,001–100,000 (USD 2640–3300)	16	3.9
	Above NTD 100,001 (USD 3300)	10	2.5

**Table 2:** Results for the factor loading, reliability, and validity.

Variables/Items	Standardized Factor Loading	AVE	CR	Cronbach’s α
Attitude (ATT)		0.706	0.905	0.858
1. Do you think that dishes prepared using molecular cooking techniques can be safely consumed?	0.839 ***			
2. Do you think that molecular cuisine tastes better than traditional cuisine?	0.862 ***			
3. Do you think that the quality of molecular cuisine is higher than that of traditional cuisine?	0.868 ***			
4. Do you think that the sources of ingredients and cooking methods used in molecular cuisine can reduce food waste?	0.789 ***			
Subject norms (SN)		0.721	0.885	0.806
1. When choosing to enjoy the molecular cuisine, do you consider your family’s opinions?	0.860 ***			
2. When choosing to enjoy the molecular cuisine, do you consider your friends’ opinions?	0.899 ***			
3. When choosing to enjoy molecular cuisine, do you refer to information from the media?	0.784 ***			
Perceived behavioral control (PBC)		0.780	0.914	0.859
1. Do you think molecular cuisine is a common cooking technique and dish?	0.884 ***			
2. Do you think that molecular cuisine is easy to acquire?	0.907 ***			
3. Do you think you can afford molecular cuisine?	0.858 ***			
Utilitarian value (UV)		0.667	0.857	0.744
1. Do you think that the style of molecular cuisine is convenient to enjoy?	0.802 ***			
2. Do you think the portion size of molecular cuisine can fill you up?	0.830 ***			
3. Do you think the ingredients in dishes prepared with molecular cooking techniques are beneficial to health?	0.818 ***			
Hedonic value (HV)		0.703	0.922	0.893
1. Do you think that the dining experience of molecular cuisine makes you feel pleasant?	0.875 ***			
2. Do you think the process of enjoying molecular cuisine makes you feel relaxed?	0.842 ***			
3. Do you think that the cooking techniques and dining process of molecular cuisine provide you with enjoyment?	0.814 ***			
4. Do you think the services and dishes provided by molecular cuisine restaurants make you feel satisfied?	0.856 ***			
5. Compared to traditional cuisine, do you think molecular cuisine offers a higher entertainment value?	0.804 ***			
Product novelty (PV)		0.814	0.946	0.924
1. Do you think that the type of dish in molecular cuisine is something you have never seen before?	0.900 ***			
2. Do you think that the cooking techniques of molecular cuisine are something you have never seen before?	0.893 ***			
3. Do you think that the dining experience of molecular cuisine is something you have never experienced before?	0.909 ***			
4. Do you think that the way molecular cuisine is enjoyed is something you have never experienced before?	0.906 ***			
Sensory stimulation (SS)		0.729	0.931	0.907
1. Do you think that the appearance of molecular cuisine is more attractive than that of regular dishes?	0.850 ***			
2. Do you think that the texture of the molecular cuisine can enhance deliciousness?	0.850 ***			
3. Do you think that the aroma emitted by molecular cuisine is better at showcasing the dish’s characteristics than that of traditional cuisine?	0.883 ***			
4. Do you think that the flavors of molecular cuisine are more unique than those of traditional cuisine?	0.856 ***			
5. Do you think the sounds made during cooking or consumption of molecular cuisine are more appetizing than those of traditional cuisine?	0.828 ***			
Behavioral intentions (BI)		0.738	0.918	0.879
1. Do you think you would recommend molecular medicine to others?	0.869 ***			
2. Do you think you would post pictures of your enjoyment of molecular cuisine on social media?	0.828 ***			
3. If given this chance, would you be willing to enjoy molecular cuisine again?	0.904 ***			
4. Can you predict what kind of dish you would choose if you were to enjoy molecular cuisine?	0.833 ***			

Note: CR: composite reliability; AVE: average variance extracted. *** *p* < 0.001.

**Table 3:** Correlation coefficients of the measurement model and the square root of AVE.

	Mean	Standard Deviation	UV	HV	ATT	SN	PBC	PN	SS	BI
UV	4.50	1.14	**0.817**							
HV	4.94	1.01	0.541 **	**0.838**						
ATT	4.72	1.04	0.635 **	0.750 **	**0.840**					
SN	4.86	1.08	0.425 **	0.521 **	0.504 **	**0.849**				
PBC	4.21	1.30	0.688 **	0.529 **	0.692 **	0.409 **	**0.883**			
PN	4.75	1.25	0.384 **	0.389 **	0.409 **	0.397 **	0.299 **	**0.902**		
SS	4.88	1.07	0.550 **	0.778 **	0.768 **	0.514 **	0.589 **	0.434 **	**0.854**	
BI	4.73	1.16	0.646 **	0.784 **	0.770 **	0.511 **	0.719 **	0.416 **	0.791 **	**0.859**

Note: The values on the diagonal (bold font on the slash) represent the square root of the average variance extracted (AVE) of the latent variables. UV: utilitarian value; HV: hedonic value; ATT: attitude; SN: subjective norms; PBC: perceived behavioral control; PN: product novelty; SS: sensory stimulation; BI: behavioral intention. ** *p* < 0.01.

**Table 4:** Model fit test.

Statistic	Recommended Value	Obtained Value	Meets Standard
χ^2^/dƒ	<3	1207.841/406 = 2.98	Yes
SRMR	<0.08	0.087	No
RMSEA	≤0.05 (marginal fit) 0.05–0.08 (good fit) 0.08–0.10 (moderate fit) >0.10 (poor fit)	0.070	Good fit
NFI	>0.9	0.879	No
CFI	>0.9	0.916	Yes
IFI	>0.9	0.916	Yes
TLI	>0.9	0.903	Yes
PNFI	>0.5	0.767	Yes
PCFI	>0.5	0.799	Yes

Note: Standardized RMSR (SRMR), root mean square error approximation (RMSEA), normalized fix index (NFI), comparative fit index (CFI), incremental fit index (IFI), Tucker–Lewis index (TLI), parsimonious normed fix index (PNFI), and parsimonious comparative fit index (PCFI).

**Table 5:** Results of the path analysis and confirmation of hypotheses.

Hypothesized Paths	Unstandardized Coefficient	Standardized Coefficients	S.E.	C.R.	*p*	Verification Results
H1: UV → ATT	1.033	0.414 ***	0.278	5.578	<0.001	Supported
H2: HV → ATT	2.041	0.817 ***	0.185	7.331	<0.001	Supported
H3: ATT → BI	0.573	0.641 ***	0.100	5.753	<0.001	Supported
H4: SNs → BI	0.039	0.017	0.039	0.366	<0.001	Unsupported
H5: PBC → BI	0.834	0.374 ***	0.834	5.050	<0.001	Supported
H6: PN → BI	0.096	0.043	0.100	0.965	<0.001	Unsupported
H7: SS → BI	1.109	0.497 ***	0.197	5.644	<0.001	Supported

Note: UV: utilitarian value; HV: hedonic value; ATT: attitude; SN: subjective norms; PBC: perceived behavioral control; PN: product novelty; SS: sensory stimulation; BI: behavioral intention. *** *p* < 0.001.

## Data Availability

The original contributions presented in the study are included in the article; further inquiries can be directed to the corresponding author.
